# Predicting the potential geographical distribution of parasitic natural enemies of the Dubas bug (*Ommatissus lybicus* de Bergevin) using geographic information systems

**DOI:** 10.1002/ece3.4286

**Published:** 2018-07-23

**Authors:** Khalifa M. Al‐Kindi, Ali K. Al‐Wahaibi, Paul Kwan, Nigel R. Andrew, Mitchell Welch, Mohammed Al‐Oufi, Zakariya Al‐Hinai

**Affiliations:** ^1^ School of Science and Technology University of New England Armidale NSW Australia; ^2^ Department of Crop Sciences College of Agricultural and Marine Sciences Sultan Qaboos University Muscat Sultanate of Oman; ^3^ UNE Centre of Excellence for Behavioural and Physiological Ecology University of New England Armidale NSW Australia; ^4^ Ministy of Agriculture and Fisheries (MAF) Muscat Oman

**Keywords:** *Aprostocetus* nr. *beatus*, *Bocchus hyalinus*, Dubas bug, GIS, natural enemies, *Pseudoligosita babylonica*, spatial statistical analysis

## Abstract

The Dubas bug (*Ommatissus lybicus* de Bergevin) is a pest species whose entire life cycle occurs on date palms, *Phoenix dactylifera* L, causing serious damage and reducing date palm growth and yield. *Pseudoligosita babylonica* Viggiani*, Aprostocetus* nr. *Beatus,* and *Bocchus hyalinus* Olmi are very important parasitic natural enemies of *Ommatissus lybicus* in northern Oman. In this study, random farms were selected to (a) model the link between occurrences of the *Pseudoligosita babylonica*,* Aprostocetus* nr *beatus,* and *Bocchus hyalinus* (dependent variables) with environmental, climatological, and Dubas bug infestation levels (the independent variables), and (b) produce distribution and predictive maps of these natural enemies in northern Oman. The multiple *R* ^2^ values showed the model explained 63%, 89%, and 94% of the presence of *P. babylonica, A. *nr *beatus,* and *Bocchus hyalinus,* respectively. However, the distribution of each species appears to be influenced by distinct and geographically associated climatological and environmental factors, as well as habitat characteristics. This study reveals that spatial analysis and modeling can be highly useful for studying the distribution, the presence or absence of Dubas bugs, and their natural enemies. It is anticipated to help contribute to the reduction in the extent and costs of aerial and ground insecticidal spraying needed in date palm plantations.

## 1 INTRODUCTION

Widespread Dubas bug (*Ommatissus lybicus* de Bergevin) infestations affect several Middle Eastern and North African countries, resulting in substantial damage to date palms (*Phoenix dactylifera* L) in some of these countries (El‐Haidari, 1982; Waller & Bridge, 1978). Dubas bugs (DBs) have been considered a major economic threat and presently affect palm growth and yields in Oman, Iraq, Iran, Pakistan, and the United Arab Emirates. Indeed, DBs have been identified as a primary reason for the decline in date production in Oman, on account of the total area infested and the large‐scale crop losses that resulted (Al‐Yahyai & Khan, 2015).

**Figure 1:**
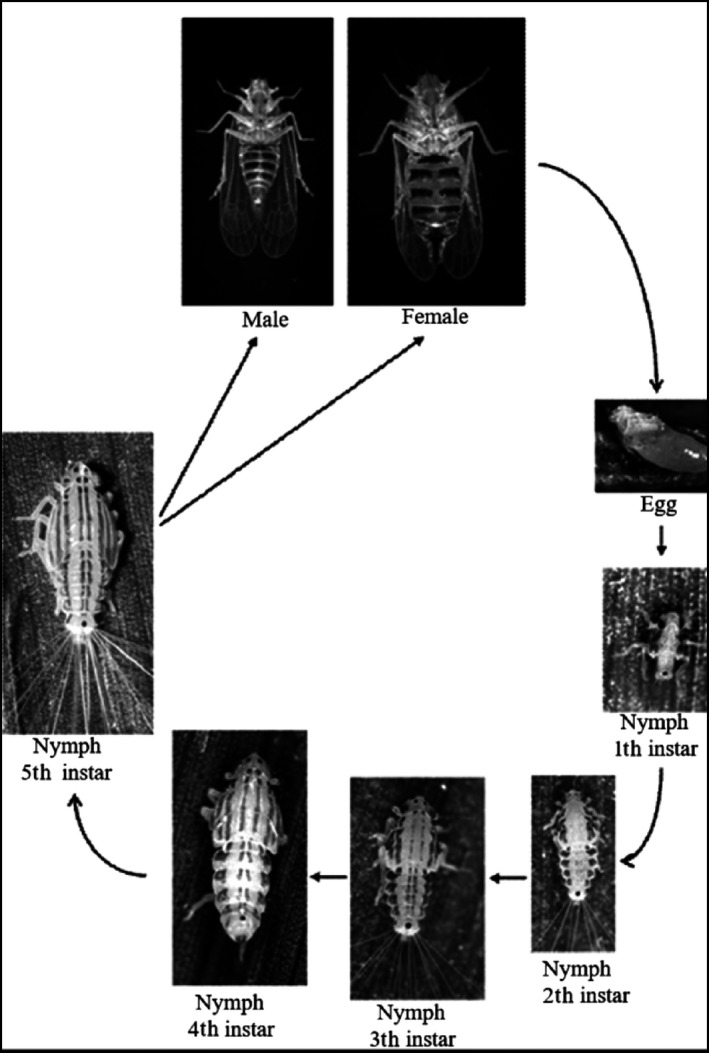
Dubas bug, *Ommatissus lybicus* life cycle (Al‐Khatri, 2011)

DBs are found on leaflets, spines, rachis, and fruit bunches of the date palms and cause direct and indirect damages to palm trees (Figure 1). The direct damage arises when the nymphs and adults feed by sucking sap from leaflets and rachis during the spring and autumn generations (Aldryhim, 2008; El‐ Shafie, Peña, & Khalaf, 2015). Direct damage also results from injury to tissue due to egg laying activity (Bagheri, Fathipour, Askari‐Seyahooei, & Zeinolabedini, 2016; Fathipour, Bagheri, Askari‐Seyahooei, & Zeinolabedini, 2018; Khalaf & Khudhair, 2015). Indirect damage comes from the presence of honeydew (sticky liquid excretion), which allows dust accumulation and sooty mould growth, causing the possible deterioration of the palm fruits and other crops that are cultivated underneath the palm trees. The copious feeding of DBs weakens the tree, and the honeydew's accumulation of dust and mould reduces photosynthesis and other physiological processes on the fronds' surfaces, which then become chlorotic after several months (Bagheri, Fathipour, Askari‐Seyahooei, & Zeinalabedini, 2017; Klein & Venezian, 1985; Mokhtar & Al Nabhani, 2010). A high DB population can cause a date palm tree to lose its vitality, leading to a decline in its productivity (Thacker, Al‐Mahmooli, & Deadman, 2003).

Controlling DBs requires the effort and money of several countries worldwide, including the Sultanate of Oman (Thacker et al., 2003). Since DBs were first recorded in Oman in 1962, activities aiming to control DB infestations have focused on the use of insecticides via annual ground and aerial sprayings. However, the control of DBs using insecticides has led to many problems. The authorities have mainly and increasingly relied on them to manage this pest, a process that costs the government large sums of money every year (Al‐Zadjali, Abd‐Allah, & El‐Haidari, 2006). From 1993 to 2011, about 523 tonnes of insecticides were used in aerial applications to control DB infestations, at an estimated cost of $18.5 million (Al‐Khatri, 2011). In 2016, the Omani government spent $1,550,000 and $395,630 for the spring and autumn generations, respectively, to control DB infestations (direct communication with the Plant Protection Department, Ministry of Agriculture and Fisheries, Oman).

Insecticides have been banned in many countries including Oman because of their perceived negative environmental effects, such as the pollution of water resources; deterioration of human health; and the resulting reductions in populations of nontarget species, particularly the natural enemies of DB and because of the relatively high application cost (Ansari, Moraiet, & Ahmad, 2014). Studies have shown that some insecticide residues can persist on date palm fruit for up to 2 months after application (Khan, Azam, & Razvi, 2001), which poses a high risk to both humans and animals. Moreover, aerial spraying is difficult to conduct on farms located within mountains, owing to high elevations and valley features (Al‐Kindi, Kwan, Andrew, & Welch, 2017a). Thus, chemical control measures have resulted in limited success in Oman, where DBs continue to pose a major challenge to the date palm industry.

The natural enemies of DB consist of a range of predatory, parasitic, and pathogenic species (Howard, 2001). Of the parasitic species, there are three relatively specific parasitoid wasp species that are present in Oman. Due to their specificity, they may be highly important factors for regulating the population of the DB. Two of these species attack the egg stage, while the third attacks the nymphal and adult stages. Relatively few studies exist on these species, and most of these have focused on the internal egg parasitoid *Pseudoligosita babylonica* Viggiani (Hymenoptera: Trichogrammatidae). This species has been recorded in Iraq, Oman, Iran, Saudi Arabia, and Yemen (Al‐Khatri, 2011; El‐Shafie, 2012; Hassan, Al‐Rubeai, Al‐Jboory, & Viggiani, 2004; Hubaishan & Bagwaigo, 2010). It may also be present in other countries where the DB exists. It is a small, stout, yellowish wasp measuring 0.7 mm in length that parasitises and kills DB eggs (Hubaishan & Bagwaigo, 2010). Each parasitoid wasp develops inside a single DB egg. A significantly higher proportion of date palm leaflets, compared to interleaflet areas (the frond parts between leaflets) showed signs of egg parasitism by this wasp (AKA, unpublished data). According to Al‐Khatri (2011), *P. babylonica* has three generations during each of the spring and autumn generations of the DB: a pre‐DB egg hatching generation, a generation coinciding with DB egg hatching, and a post‐DB egg hatching generation. Al‐Khatri (2011) studied the biology and ecology of *P. babylonica* in Oman and stated that this egg parasitoid could be considered a potential biological control agent of DB.

The second parasitoid associated with the eggs of DB is *Aprostocetus* nr. *beatus* (Eulophidae: Hymenoptera) (Kinawi, 2005). The mature larva of this wasp has been observed to be an external feeder on one or more eggs of the DB within the midrib area of date palm leaflets (AKA, unpublished data), but younger larvae could act as internal egg parasitoids, as demonstrated in the case of *A. . beatus* attacking eggs of other planthoppers and leafhoppers in other parts of the world (Carnegie, 1975, 1980; Kosheleva & Kostjukov, 2014).

The nymph‐adult parasitoid *Bocchus hyalinus* Olmi (Hymenoptera: Dryinidae) has been recorded as present in Oman, the United Arab Emirates (UAE), and Kenya (Olmi, Copeland, & Guglielmino, 2015). Olmi (1998) described this wasp more than a decade ago, based on a female specimen collected in Oman. This small wasp species develops in the nymphs and adults of the DB, producing a dark pouch or sac that protrudes from the abdomen of its host. Although both sexes are winged, the female's body is generally yellowish‐orange, measuring 2.8–3.8 mm in length, whereas the male is black to brown and measures 1.5–2.25 mm (Olmi, 2008, 2009).

Although a few studies exist on the above parasitic natural enemies of DB (e.g., *P. babylonica, A. *nr. *beatus* and *B. hyalinus*) (Al‐Khatri, 2011; Hassan et al., 2004; Hubaishan & Bagwaigo, 2010), none have applied geographic information system (GIS) and spatial analyses to these species in the Sultanate of Oman. In addition, the effective biological control of a pest requires biological and ecological knowledge: host plant(s), host insects, biological control agent(s), and the locations (areas) where the biological control agent is going to be used. No focused research exists on the spatial and temporal distributions of these natural enemies and their relationships with environmental, climatological, and resource availability conditions. Practical tools and approaches are needed for mapping, analyzing and, more importantly, predicting the distribution of these important DB natural enemies and for understanding the interactions between these enemies and environmental and climatological factors, so that decision makers may improve the management of DB infestations on a large scale.

The main objective of this study was to investigate how the environmental, climatological, and DB infestation level variables impact populations of *P. babylonica, A. *nr. *Beatus,* and *B. hyalinus* in northern Oman. We considered environmental (e.g., elevation, slope, aspect or direction of slope, soil, water type and distance to streams), climatological (e.g., temperature, humidity, rainfall, and wind direction), and DB infestation level variables and incorporated them into a GIS platform to evaluate which combinations of variables are associated with these natural enemies of DBs. This study will also allow for the prediction of the future distribution of populations of these three potential biological control agents of DB in Oman.

## 2 MATERIALS AND METHODS

### 2.1 Study area

**Figure 2:**
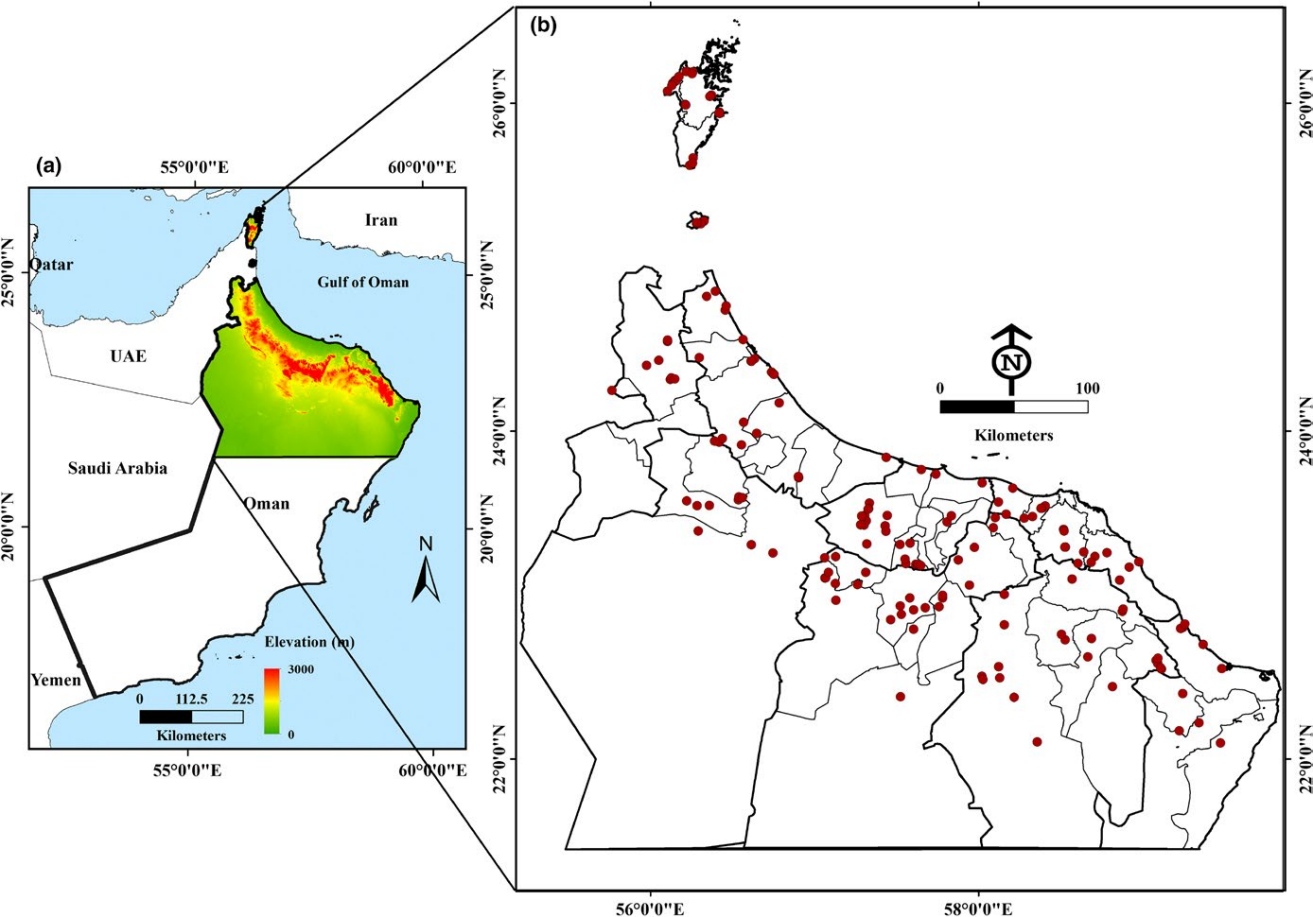
The study areas: (a) location and topography, (b) nine governorates in the Sultanate of Oman, with date palm plantations sampled for DB and its natural enemies highlighted in red

Oman covers an area of 309,500 square kilometers and extends from 16°40′N to 26°20′N, and 51°50′E to 59°40′E. It occupies the south‐eastern corner of the Arabian Peninsula. It has 3,165 km of coastline, extending from the Strait of Hormuz in the north to its border with the Republic of Yemen in the south. The coastline faces three bodies of water, namely the Arabian Sea, the Persian Gulf (also known as Arabian Gulf), and the Gulf of Oman (Figure 2).

To the west, Oman is bordered by the UAE and the Kingdom of Saudi Arabia. Mountainous areas account for 15% of all land area, while desert plains, sandy areas, and coastal zones cover 77% of the land area. The remaining land area is covered by agricultural land. According to the 2004–2005 soil survey conducted by the Ministry of Agriculture and Fisheries (MAF), in the Sultanate of Oman, 22,230 km^2^ (equivalent 2.223 million ha) are optimal for agricultural activity, which represents roughly 7.5% of the country's total land area. Oman's location provides favorable conditions for agriculture development. Land devoted to agriculture uses accounted for an economic output equal to 14.6% of the GDP in 2008 (Al‐Kindi, Kwan, Andrew, & Welch, 2017d).

The elevation ranges in the study zone from 0 to 2,994 m above sea level, and the soil contains five soil‐type categories: clay, gravel, loam, rock, and sand. The water in the area can be categorized into three classes based on total dissolved content: brackish water, freshwater, and saltwater (Al‐Kindi et al. 2017a). Oman has an arid climate, receiving <100 mm of rain per year; however, the mountainous parts of the country enjoy higher precipitation levels. As one of the independent variables, DB infestations occur where palm trees are concentrated; therefore, in the present study, we focused on northern Oman, the area from latitude 26°50ʹN to 22°26ʹN and from longitude 55°50ʹE to 59°50ʹE (see Figure 2). The study region is approximately 114,336 km^2^ in area. The date palm plantations are denser in the coastal plains and many interior places in the study area (Al‐Busaidi & Jukes, 2015). The area covered by this study contains different levels of DB infestation. Higher levels of DB populations have resulted in direct and indirect damage to infested palm trees and nearby trees (Al‐Kindi, Kwan, Andrew, & Welch, 2017c).

### 2.2 Data collection and analysis of DBs

#### 2.2.1 Sampling periods and sites

Date palm materials and insect specimens were obtained during four periods: summer (June–August) 2009, spring (March–June) 2010, spring (March–April) 2011, and spring (April) 2015. The first round of sampling, in summer 2009, targeted the DB's egg stage, so no nymphs or adults were present in the samples. The spring of 2010, 2011, and 2015 rounds of sampling targeted the nymphal and adult stages, which tended to be higher in number during the spring generation than during the fall generation. Sites visited during the first round of sampling included all Oman governorates (Muscat, Al‐Batinah North, Al‐Batinah South, Musandam, Al‐Buraymi, Al‐Dhahirah, Al‐Dakhiliyah, Ash‐Sharqiyah North, Ash‐Sharqiyah South, Al‐Wusta and Dhofar).

The second round of sampling included all governorates except Al‐Wusta and Dhofar. The third included sites in Muscat, Ash‐Sharqiyah South, and Dhofar, while the fourth round involved only one site in Al‐Dakhiliyah. Al‐Wusta was not visited during the second, third or fourth rounds because the first round of sampling revealed no records of DB there. All sites visited in each governorate were chosen at random. They included, for the most part, farm sites (cultivated or neglected) in villages and towns or, more rarely, aggregations of wildly growing date palms in wadis. Sites were marked geographically using GPS device (Garmin) to give positional and elevation data, and site names were recorded.

#### 2.2.2 Sampling of DB eggs

Three to five trees were selected randomly at each site. In most cases, samples were taken from shorter trees, those up to 2 m in height (growing point height). Fronds of these trees were reachable from the ground, making them easy to examine and cut. One middle‐aged green frond was cut from each tree. All fronds were examined for the presence of new DB eggs. At farm sites with tall trees, a farm attendant/laborer was asked to climb each tree to collect one green frond. Each collected frond was then cut into 3–6 pieces, which were placed in large trash bags. In some cases, leaflets were excised from the frond (using pruning scissors) and then placed in bags. Trash bags with frond material were placed in a shaded area in the field and then moved inside a large cool box. Finally, they were transported to the entomology laboratory at College of Agricultural and Marine Sciences, Sultan Qaboos University.

#### 2.2.3 Sampling of DB nymphs and adults

Sampling was performed on relatively short date palms of up to 2 m in height. At least five trees were sampled at each site, but when the DB populations were low, up to ten trees were examined. Nymphs and adult DBs were sampled using three methods. When populations were relatively large, suction was applied using handheld vacuum machines (Black & Decker car vacuum, and Bio‐quip custom‐made vacuum). DBs collected via a vacuum machine from a particular site were pooled inside a large jar. When populations were relatively low, one of the two following methods was used: Removal of leaflets infested with nymphs and adults using pruning scissors, and then, leaflets were placed inside a large jar; or shaking of the leaflets to dislodge the insects into a large jar. The field samples were taken roughly between 8 a.m. and 11 a.m. as this is when the insects are feeding and not moving off the leaflets due to excessive heat exposure.

#### 2.2.4 Processing egg samples in the laboratory

If not already performed in the field, leaflets were separated from the rest of the fronds in the lab. All leaflets and frond pieces were then checked for new eggs. Leaflets and frond pieces with new eggs were retained, and the rest were discarded. The apical parts of leaflets were cutoff, and the leaflets were placed into large, 5‐litre jars with a small amount of water at the bottom. Some space was left in the jar between leaflets, jar to allow for sufficient aeration. Leaflets from each farm site were combined together, but in cases with a large number of leaflets, they were distributed in more than one jar. Frond pieces were placed in 5‐litre jars in a similar manner to that described above for leaflets. All jars were labeled with site information and the collection date. Frond material was incubated for 2 months at an average, minimum, and maximum temperatures of 22.29, 19.42, and 27.52°C respectively. The average, minimum, and maximum humidity were 49.94%, 27.1%, and 66.1%, respectively.

Frond pieces and leaflets from each site were checked twice weekly for insects, including potential emerging parasitoids. Interleaflet frond areas and leaflets with observed adult parasitoids were marked with red ink and kept separate from the rest of the frond material, to make it easier to follow up on the progress of parasitism of eggs. The material was also checked for the emergence holes of parasitoids in or around DB eggs. Emerged or observed stages of parasitoids were photographed.

#### 2.2.5 Processing of nymph‐adult samples in the laboratory

For a particular site, all collected nymphs and adults were combined in small plastic vials containing 80% ethanol as a preservative. These vials were labeled to indicate each sample's site and date and were stored at room temperature for later assessment. The presence of nymphal‐adult parasitism was determined in a particular site by examining sampled nymphs and adults and checking for the presence of a characteristic dark (brown‐black) sac, indicative of parasitism, on the side of the abdomen of each DB.

### 2.3 Data collection and spatial analysis of natural enemies

#### 2.3.1 Parasitoid data

Although the study samples came from many governorates, data used in GIS analysis are based only on samples from nine governorates in northern Oman: Al‐Dakhiliyah, Al‐Dhahirah, Al‐Batinah (North and South), Ash‐Sharqiyah (North and South), Al‐Buraymi, Musandam, and Muscat. The natural enemies' data of parasitoids *P. babylonica, A. beatus,* and *B. hyalinus* were based on collections made at 168 locations. All collected data were then converted to a GIS shapefile using ArcGIS 10.4 (ESRI, Redlands, CA) for mapping and spatial analyses. The shapefile data were used to map the absence and presence of *P. babylonica*,* A. beatus,* and *B. hyalinus* in the study area.

Data on DB infestations and their impact were determined by observations of their prevalence in palm trees from 2009 through 2015 based on data obtained from the Ministry of Agricultural and Fisheries (MAF), Oman. These data comprised spatial coordinates (sites' longitudes and latitudes), governorates and DB infestation levels. First, we classified the DB infestation levels into four groups (very low, low, moderate, and high) in ArcGIS as follows: very low infestation, 0–5 nymphs per leaflet; low, 5–7 nymphs per leaflet; moderate, 8–9 nymphs per leaflet; and high infestation, 10 or more nymphs per leaflet (Al‐Kindi et al., 2017c). In addition, we calculated the average levels of DB infestation over two seasons (spring and autumn generations) from 2009 to 2015. By linking attribute identifications of the pixels with continuous datasets (environmental, climatological, and DB infestation levels), we displayed the presence of the natural enemy species in the GIS rasters.

#### 2.3.2 Environmental and climatological data

To examine the environmental and climatological variables associated with the distribution of *P. babylonica*,* A. beatus,* and *B. hyalinus*, data on elevation, slope, aspect, hillshade, soil types, water type, distance to streams (wadis), precipitation, temperature, humidity, wind direction, and wind speed were obtained for each collection location of natural enemies and compiled into a database. First, we obtained elevation and derived slope, aspect, hillshade, and rivers (dry valleys) for each site by importing the digital elevation model (DEM) map into ArcGIS 10.4 and extracting the underlying value (in meters). Second, the Euclidean distance method (i.e., surface analysis) was used to calculate the distance from the observation points using the “wides” feature (Al‐Kindi et al., 2017d).

The vector GIS dataset, including governorate boundaries and shapefiles, was obtained from the National Centre for Statistics & Information, Oman. The water type dataset (e.g., freshwater, saltwater, and brackish water) was obtained from the Ministry of Regional Municipalities and Water Resources (MRMWR), Oman. The climate data, such as temperature, humidity, wind speed, and wind direction, were obtained from the Directorate General of Meteorology, Oman. The averages of temperature, humidity, wind speed, and wind direction were calculated for 22 weather stations in the study area over the period 2009–2015. The average rainfall from 2009 to 2015 was calculated for 120 weather stations in the study area, using data obtained from MRMWR. Interpolation techniques, in combination with GIS, were used to interpolate the weather spatially. The climate in each parasitoid sampling location was estimated by interpolating the data from all enrolled weather stations, using the inverse distance weighting and spline methods (Abatzoglou, 2013). Finally, we linked climatological, environmental, and DB infestation levels data with the presence/absence of *P. babylonica, Aprostocetus* nr. *Beatus,* and *B. hyalinus*.

### 2.4 Data and spatial analysis

To understand the factors behind observed spatial patterns or to predict spatial distributions, regression analysis methods are useful for modeling, examining, and exploring these relationships. In this study, ordinary least square (OLS) and geographically weighted regression (GWR) for multiple predictions in ArcGIS 10.4^®^ (Wooldridge, 2003) were used to generate prediction maps and modeled the relationships (Braun & Oswald, 2011) between the dependent (*P. babylonica, Aprostocetus* nr*. Beatus,* and *B. hyalinus*) variables and independent (environmental, climatological, and DB infestation levels) variables. The independent variables included DB infestation levels, elevation, slope, aspect, hillshade, soil type, water type, distance to streams (wadis), precipitation, temperature, humidity, wind speed, and wind direction.

First, the OLS model was used to compute a single coefficient to represent both positive and negative relationships between the dependent and independent variables. This tool automatically checks for multicollinearity and computes standard errors and model significance indices that are robust to heteroscedasticity. In addition, the output attribute data from the OLS and GWR for multiple regression analysis, including residual, coefficients, and *p*‐values, were used to create maps, tables, and figures showing the model's predictions of *P. babylonica, A. *nr *beatus,* and *B. hyalinus*, including predictions of area of overproduction and underproduction of these parasitic species.

## 3 RESULTS

### 3.1 Natural enemies mapping

**Figure 3:**
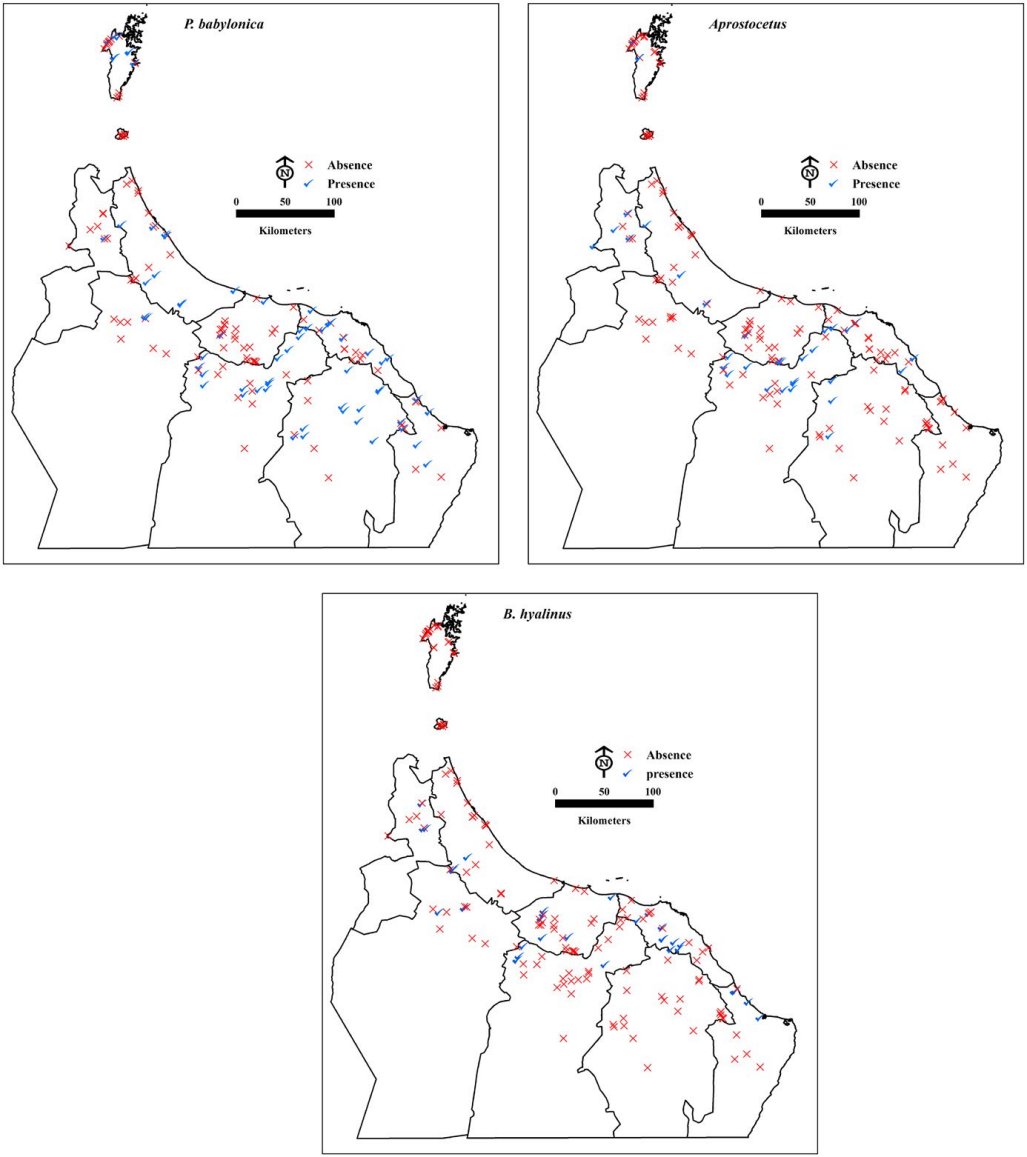
Points showing observed the presence (red) and absence (blue) of *P. babylonica, A. *nr. *beatus* and *B. hyalinus* in the study area

The most prevalent parasitoid species was *P. babylonica*, which was found at 66 sites, followed by *Aprostocetus* nr. *beatus* at 35 sites and *B. hyalinus* at 33 sites (see Figure 3). Of the *P. babylonica*,* Aprostocetus,* and *B. hyalinus* data, a total of 168 collection sites returned information. The collection sites were located predominately in nine geographical governorates in northern Oman. The distribution of *P. babylonica* occurred in all nine governorates but varied across the study area. *Aprostocetus* nr. *beatus* distributions were found primarily in seven governorates, being absent in Al‐Dhahirah and Ash‐Sharqiyah South. By contrast, *B. hyalinus* distributions were found in seven governorates, but not in Musandam or Ash‐Sharqiyah North (Figure 3).

### 3.2 Nearest neighbour statistical analysis

The results of our nearest neighbour statistical analysis, in which the nearest neighbour ratios were 0.520, 0.636, and 0.689, respectively, showed that the expected mean (EM) distance (or spacing) of *Aprostocetus* nr*. beatus, B. hyalinus,* and *P. babylonica* distributions were, respectively, higher than the observed mean (OM), with the difference less than zero (a negative number). These results indicated that the distributions of *Aprostocetus* nr*. beatus, B. hyalinus,* and *P. babylonica* were largely clustered (see Table 1).

**Table 1:** Mean nearest neighbour distribution of *A. *nr*. beatus, B. hyalinus,* and *P. babylonica*

	Observed mean distance (km)	Expected mean distance (km)	Nearest mean ratio	*Z*‐score	*p*‐Value
*Aprostocetus* nr*.beatus*	12.14	23.33	0.520	−5.344533	0.00001
*B. hyalinus*	12.75	20.04	0.636	−3.263810	0.00109
*P. babylonica*	14.26	20.67	0.689	−4.326245	0.00015

### 3.3 Spatial relationships modeling analysis

The model variables that best explain the occurrence of *P. babylonica*,* A. *nr*. Beatus,* and *B. hyalinus* in the study area, along with their variance inflection factors (VIF), are shown in Table 3. VIFs are based on tests designed to measure whether two or more factors are telling the same story (O'brien, 2007). The idea is that any factor that has a value of higher than 7.5 should be removed from consideration. The preliminary steps in the OLS analysis, multicollinearity testing by mean square deviation and VIF were run separately (once per species) on the pool of 13 independent factors, which were selected to correspond to the conceptual model (see Table 2). The average VIF value in Model 1 was 1.49, followed by Model 2, at 2.11 and 1.96 in Model 3 (see Table 3). The VIF values in the three models are all well under the ESRI‐defined threshold of 7.5, confirming that these factors are not redundant (see Table 2).

**Table 2:** Best fit variables from OLS exploratory regression and their related VIF values

	Dependent variables		
	*P. babylonica* (model 1)	*A. *nr*. beatus* (model 2)	*B. hyalinus* (model 3)
Independent variables	VIF	VIF	VIF
DB infestation levels	1.97	1.91	1.93
Elevation	1.25	3.87	2.96
Slope	1.47	1.81	2.37
Aspect	1.39	1.64	1.52
Hillshade	1.31	1.94	1.99
Water type	1.63	2.37	2.67
Soil type	1.23	1.58	1.30
Temperature	1.70	2.46	1.24
Humidity	1.86	1.96	2.72
Wind direction	1.85	2.37	1.82
Wind speed	1.34	2.40	1.47
Rainfall	1.23	2.06	2.14
Distance to (wadis)	1.56	2.16	1.91

**Table 3:** *p*‐Values showing statistically significant variables

	*P. babylonica*	*A. *nr. *beatus*	*B. hyalinus*
DB infestation	+0.0001*	+0.0001*	+0.0002*
Elevation	+0.0001*	+0.0006*	+0.0001*
Slope	0.7357	0.0528	0.9168
Aspect	0.4197	0.3121	0.8772
Hillshade	0.0824	0.8771	0.5245
Water type	+0.0006*	0.3803	+0.0026*
Soil type	0.0824	0.0202	0.2538
Temperature	−0.0003*	−0.0013*	−0.0019*
Humidity	+0.0044*	−0.0001*	+0.0001*
Wind direction	+0.0025*	+0.0001*	0.0715
Wind speed	0.1500	−0.0001*	−0.0005*
Rainfall	0.0374	0.2055	+0.0001*
Wadis	0.6904	0.3144	0.0599

>Notes.

An asterisk next to a number indicates a statistically significant *p*‐value (*p *<* *0.01).

OLS and multiple regression revealed that the *P. babylonica* distribution had significant positive relationships *p*‐value (*p *<* *0.01) with DB infestation level (*R* ^2^ = 0.80), elevation (*R* ^2^ = 0.83), wind direction (*R* ^2^ = 0.64), humidity (*R* ^2^ = 0.45), and water type (*R* ^2^ = 0.48); however, significant negative correlations were found between temperature (*R* ^2^ = 0.53) and *P. babylonica* presence (Table 3).

Table 3 shows significant positive association *p*‐values (*p *<* *0.01) were found between *A. *nr*. beatus* and elevation (*R* ^2^ = 0.90), DB population (*R* ^2^ = 0.88), and wind direction (*R* ^2^ = 0.71); nevertheless, significant negative associations were found between the temperature (*R* ^2^ = 0.93), humidity (*R* ^2^ = 0.94), and wind speed (*R* ^2^ = 0.60) factors and *A. *nr*. beatus* presence. In addition, significant positive relationships were found between *B. hyalinus* and DB population (*R* ^2^ = 0.81), elevation (*R* ^2^ = 0.72), humidity (*R* ^2^ = 0.46), rainfall (*R* ^2^ = 0.74), and water type (*R* ^2^ 0.39); but significant negative relationships were found between temperature (*R* ^2^ = 0.94) and wind speed (*R* ^2^ = 0.37) on the one hand and *B. hyalinus* presence on the other hand (see Table 3).

**Figure 4:**
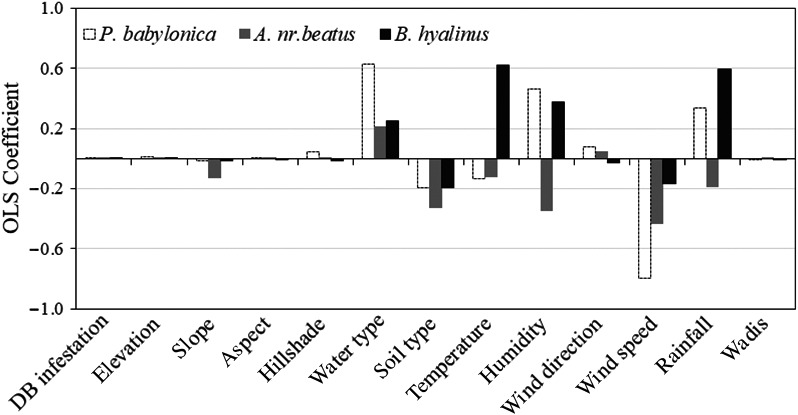
Coefficients among environmental, climatological, and DB infestation variables (independent variables) and *P. babylonica, A. *nr*. Beatus,* and *B. hyalinus* (dependent variable)

Several factors in Table 3 were significant at a confidence level of >95%, which indicates strong relationships between individual exploratory factors and the dependent variable. The coefficient values displayed in Figure 4 shows the impact of each of the variable that has the strongest correlation with *P. babylonica, A. *nr, and *B. hyalinus*, and that other variables still predicted a strong correlation with *P. babylonica, A. *nr*. Beatus,* and *B. hyalinus* distributions in the study area.

**Figure 5:**
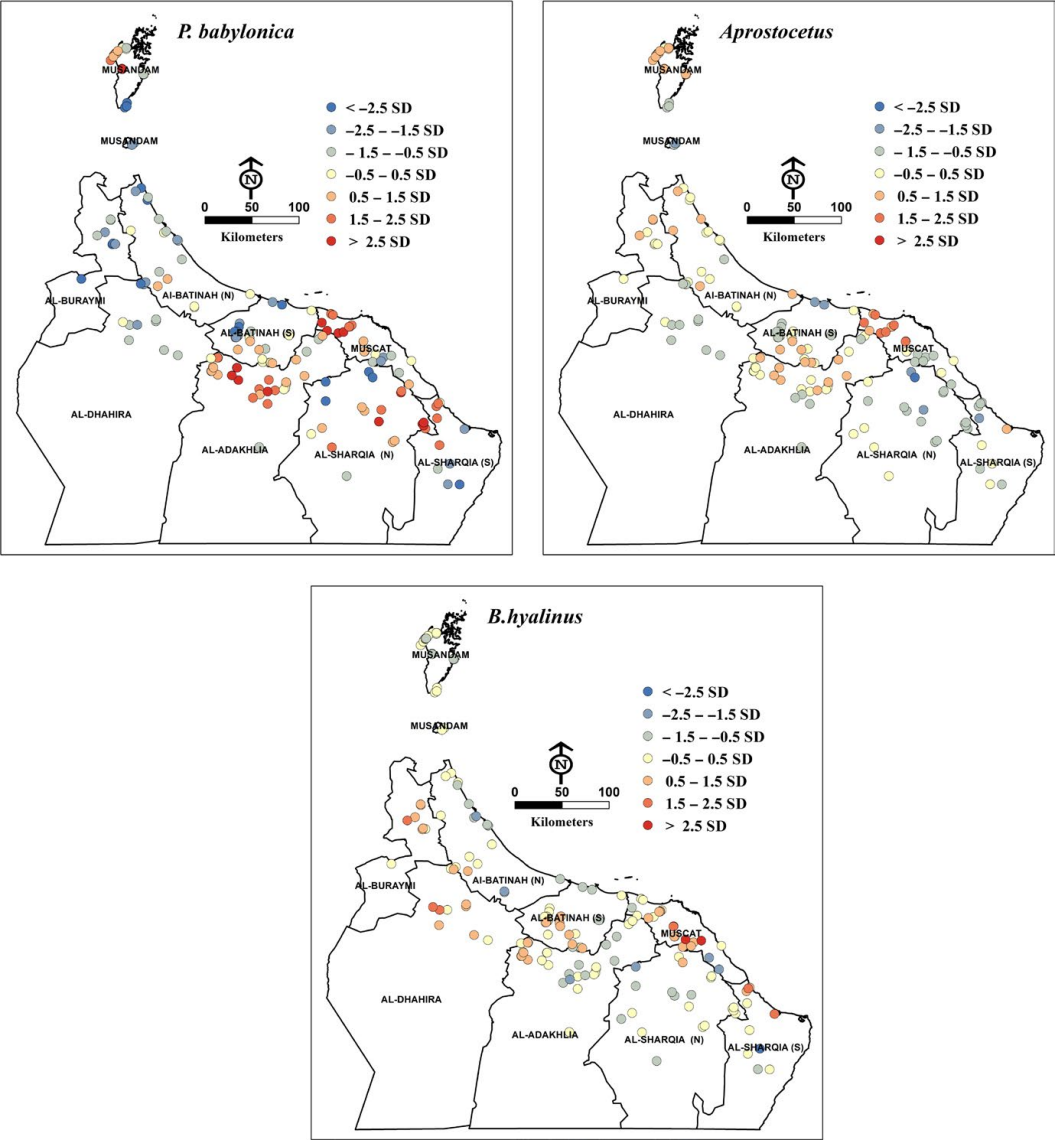
Ordinary Least Square (OLS) maps of standardized residuals showing the spatial patterns of under‐ or over‐production of the natural enemies

The significance of the models' predictions is evident in the mapping of residual standard deviations. The models produced under predictions, although it is likely that other variables could also predict strong correlation with *P. babylonica, A. *nr, and *B. hyalinus* in the study area as shown in (Figure 5). The three parasitoid models explained 63%, 89%, and 94% of the impact of environmental, climatological and DB infestation levels on *P. babylonica, A. *nr*. Beatus,* and *B. hyalinus*, respectively.

## 4 DISCUSSION

Results shown in Table 1 indicated that the expected mean distances of the species' distributions were greater than the observed mean distance. These results indicated the presence of clustered distributions of *P. babylonica, A. *nr. *Beatus,* and *B. hyalinus* in the study area. The results of the OLS regression method revealed models that confidently predict 63%, 89%, and 94% of the influence of DB infestation levels, and climatological and environmental variables on the *P. babylonica, Aprostocetus* nr*. beatus* and *B. hyalinus* presence in the study area, respectively.

Although *P. babylonica* is by far the most important natural enemy of DB, the result shows that the ability of the studied independent variables to predict the distribution of this natural enemy is relatively low (63%) compared with the two other parasitic. One possible explanation for this contrast between *P. babylonica* and the other two parasitoids involves differences in life history between the three species. *P. babylonica* is an internal egg parasitoid of DB eggs which are mostly hidden (expected for operculum or egg cap) within date palm leaf tissues. *A. *nr*.beatus* is an external egg feeder as a mature larva and possibly an internal egg feeder as a younger larva, and *B. hyalinus* feeds on nymphs and adults. A 63% explanation by the model is still quite strong, but as it is purely an internal egg parasite, the low *R* ^2^ value could be due to other biological variables that we did not take into account when developing the model.

**Figure 6:**
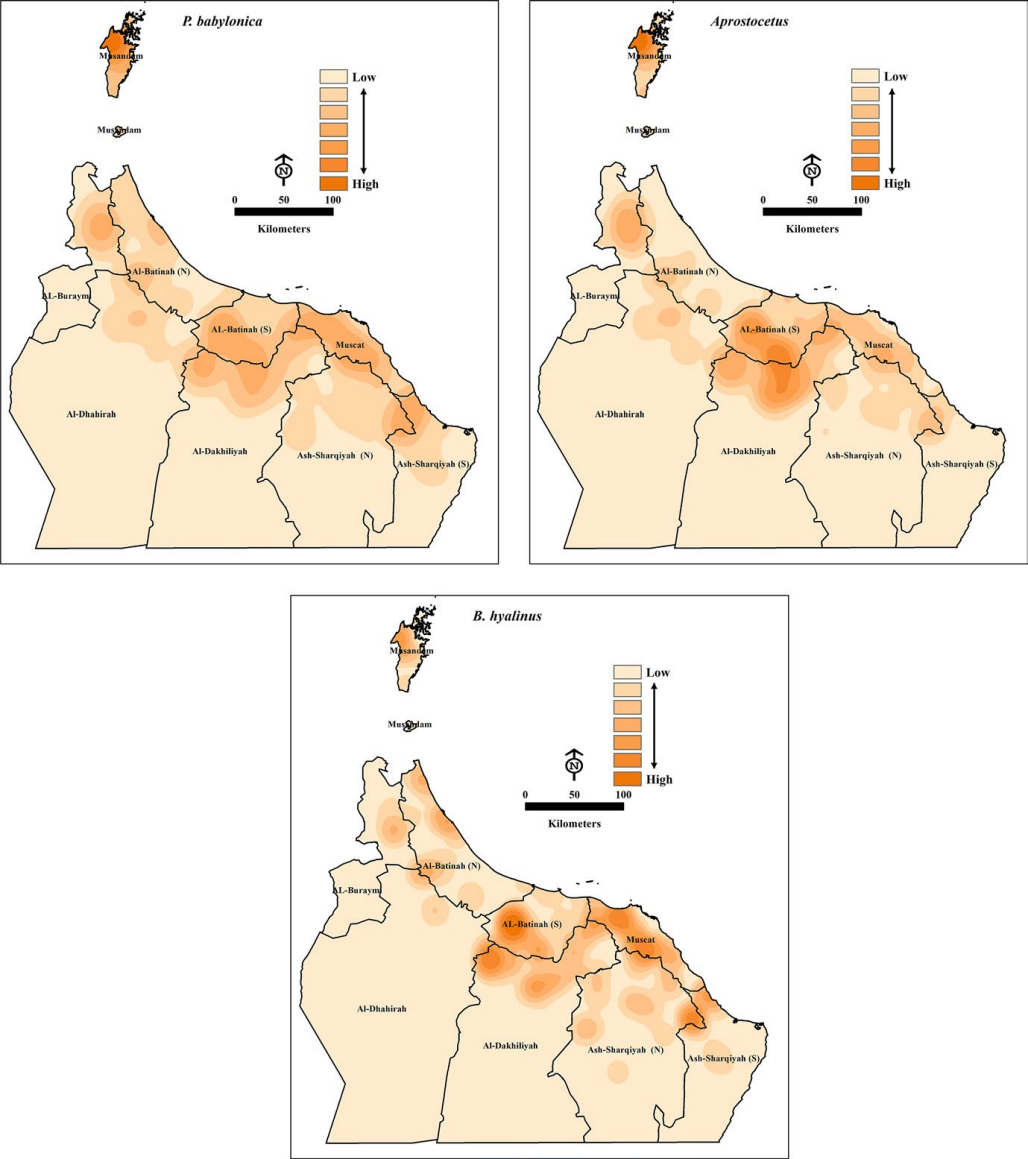
OLS models' predictions of influences of all independent variables (climatological, environmental, and DB infestations) on the presence or absence of *P. babylonica, A. *nr. *Beatus,* and *B. hyalinus* in the study area

Our environmental and climatological analyses suggested that DB infestation levels, elevation, water type, temperature, humidity, rainfall, wind speed, and wind direction drive the spatial distribution of each natural enemy, as well as differences between them. The distribution of each species is influenced by distinct and geographically associated climatological, environmental factors, and habitat characteristics. In particular, high suitability was predicted in the Al‐Dakhiliyah North, Al‐Batinah South, Ash‐Sharqiyah South (north‐west), Muscat, and Musandam (north‐west) governorates for *P. babylonica*. DB population, elevation, temperature, humidity, wind direction, and water type were important predictors for calculating the probability of occurrence of *P. babylonica*. Likewise, we found high suitability in Al‐Dakhiliyah North, Al‐Batinah South, and Musandam governorates for *A. *nr*. beatus*, where factors such as DB infestation, elevation, temperature, humidity, wind direction, and wind speed are significant predictors for calculating the probability of its occurrence. Similarly, we found high suitability in Al‐Dakhiliyah North, Al‐Batinah South, Muscat, and Ash‐Sharqiyah (north‐west), where factors such as DB infestation, temperature, humidity, rainfall, and water type were important predictors in calculating the probability of occurrence for *B. hyalinus* (see Figure 6).

**Figure 7:**
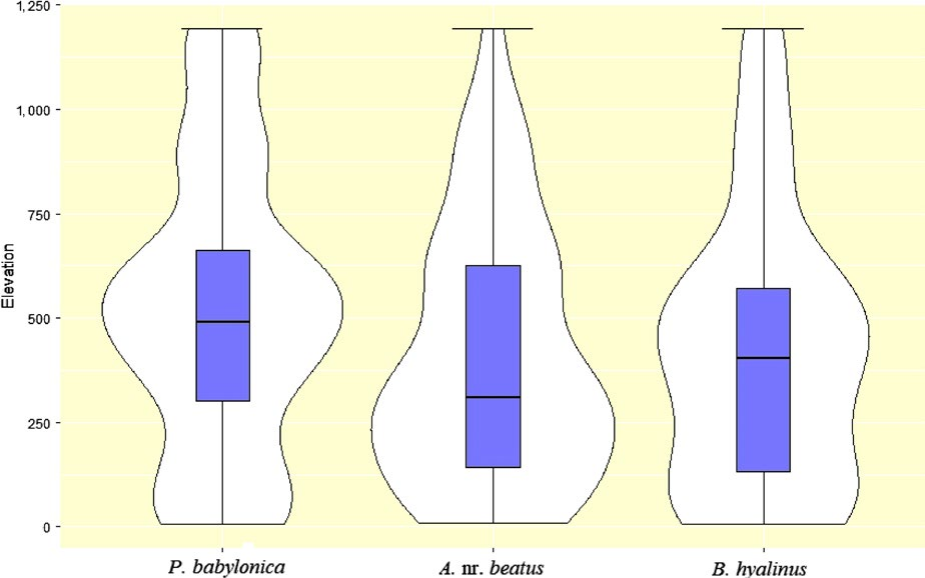
Frequency distributions of *P. babylonica*,* A. *nr. *Beatus,* and *B. hyalinus* with respect to elevation in the study area

*Pseudoligosita babylonica* occurred most frequently in elevation classes of 7–619 m and 898–918 m. It also occurred between 1,000 and 1,047 m, but in smaller proportion (mean ± *SD* = 411 ± 323 m). *A. *nr. *beatus* occurred at a high rate of frequency in the 7–600, 826–850 and 1,099–1,192 m elevations (519 ± 343 m). *Bocchus hyalinus* occurred most frequently in elevation classes of 10 –1,114 m (428 ± 309 m) (see Figure 7). The influence of elevation and DB infestation variables on the presence of *P. babylonica*,* A. *nr. *beatus* and *B. hyalinus* was concentrated in farms located between mountains and less concentrated in desert and coastal areas away from the mountains. In particular, higher infestations were more severe in mountain wadi biomes and less in the open areas.

Wind direction has a positive impact on the presence of *P. babylonica* and *A. *nr*.beatus*. Both species were found most frequently among wind direction classes between 148–180° SW and 212–277° SE. The southwest wind, which is commonly known as the “kous” in the Gulf countries, is warm and moist. This wind may bring with it viable conditions for these two species to survive in northern Oman. In contrast, we found no influence of wind direction on the *B. hyalinus*.

Temperature, relative humidity, and rainfall are critically important biotic factors that can influence insect development and the presence of insect pests and their natural enemies (Feeley, Malhi, Zelazowski, & Silman, 2012). The present study's regression analyses indicated that humidity was an important factor for *B. hyalinus, A. *nr*. Beatus,* and *P. babylonica*. We found that, as the mean daily humidity increased, so did the occurrence of *B. hyalinus* and *P. babylonica* in the study area. *P. babylonica* and *B. hyalinus* were found to be present where mean daily humidity ranged between 38% and 63%, with means of 48% and 49%, respectively. In contrast, negative significant relationships were found between temperature and *A. *nr*. beatus* and *P. babylonica* occurrence. Our results also showed negative significant relationships between wind speed and *A. *nr*. beatus* and *B. hyalinus* species. This is possible because DBs avoid extreme temperatures (both high and low), direct sunlight, and dry areas with disturbing winds (Al‐Kindi et al., 2017d).

Our results also found that higher mean rainfall increases the occurrence of *B. hyalinus*, but no correlation was found between rainfall and *A. *nr*. beatus* and *P. babylonica* in the study area. Observations on the distributions and predominance of *P. babylonica,* in a larger portion of the palm plantation in Oman, as found in this study, confirmed the suggestions that *P. babylonica* has a broad environmental range and that it is found in more locations than *Aprostocetus* nr and *B. hyalinus*.

In this study, we have analyzed the distribution patterns of the presence or absence of *P. babylonica, Aprostocetus* nr and *B. *hyalinus, which are natural enemies of the DB, in northern Oman. Identifying patterns for different sets of features is crucial for understanding the ecological processes (Al‐Kindi, Kwan, Andrew, & Welch, 2017b; Haslett, 1990) of DBs and their enemies. In general, there are two approaches of identifying patterns in geographical data. The first is to display features on a map without conducting any statistical analyses, as showing the data in a spatial format can be valuable endeavor, even without detailed analysis (see Figure 3).

The second approach is to use spatial statistics to measure the extent to which features are clustered, dispersed, or random (Getis & Ord, 1992). Each of these measures is important when comparing the patterns for different sets of features or when comparing patterns across a given area (Kozak, Graham, & Wiens, 2008). Geostatistical analyses allow us to generate optimal surfaces from sample data and to evaluate predictions, leading to better decision‐making. GIS techniques are helpful in many data analysis models, such as environmental, precision agricultural, wildlife, and ecological studies (Al‐Kindi et al., 2017d). However, the results of the present study are in the form of first approximation models. More data and ecological information on *P. babylonica, A. *nr. *Beatus,* and *B. hyalinus* could produce better results in the future. Hence, more surveys are needed to determine the distribution and density of these natural enemies for controlling DBs and for recording other enemies that can be used successfully against DBs throughout Oman. With this in mind, the relationship between natural enemies and DB infestation should be investigated prior to any planning for chemical control application. Farmers and the responsible authorities in Oman should take care of these natural enemies of DBs using alternative methods of insecticide application, to minimize impact on nontarget insects including beneficial ones such as honeybees and other pollinating insects as well as natural enemies of pest insects.

## 5 CONCLUSION

*Pseudoligosita. babylonica, A. *nr. *Beatus,* and *B. hyalinus*, along with other natural enemies, may provide effective control of DB infestations throughout Oman if their populations are not disturbed by human activities, such as the use of pesticide sprays. A greater understanding of the ways in which environmental and climatological factors determine the distribution of DBs and their natural enemies is expected to lead to new methods of predicting which areas of a palm plantation are most at risk for infestation. The targeted application of insecticides in date palm plantations to areas of high DB infestation and low occurrence of its natural enemies would control the DB infestation better than a blanket application to all plantations without regard to DB infestation level or occurrence of associated natural enemies. Advancing our knowledge of environmental and climatological factors and behavioral responses determining the spatial and temporal distribution of DBs and their parasitoids may also lead to the development of more effective IPM strategies for DBs based on treatments which are more precise, both spatially and temporally.

## CONFLICT OF INTEREST

None declared.

## AUTHORS CONTRIBUTION

K.A., P.K., and N.A. conceived of the presented idea. K.A. developed the theory and performed the computations. K.A., A.K.A., P.K., N.A., and M.W. verified the analytical methods. M.A. and Z.A. collected the samples under the supervision of A.K.A. All authors discussed the results and contributed to the final manuscript.
